# 1/*f* Noise Mitigation in an Opto-Mechanical Sensor with a Fabry–Pérot Interferometer

**DOI:** 10.3390/s24061969

**Published:** 2024-03-20

**Authors:** Andrea M. Nelson, Jose Sanjuan, Felipe Guzmán

**Affiliations:** 1 Wyant College of Optical Sciences, The University of Arizona, Tucson, AZ 85721, USA; 2 Department of Aerospace Engineering, Texas A&M University, College Station, TX 77483, USA

**Keywords:** opto-mechanics, displacement measurement, 1/*f* noise, interferometry, Fabry–Pérot cavity

## Abstract

Low-frequency and 1/*f* noise are common measurement limitations that arise in a variety of physical processes. Mitigation methods for these noises are dependent on their source. Here, we present a method for removing 1/f noise of optical origin using a micro-cavity Fabry–Pérot (FP) interferometer. A mechanical modulation of the FP cavity length was applied to a previously studied opto-mechanical sensor. It effectively mimics an up-conversion of the laser frequency, shifting signals to a region where lower white-noise sources dominate and 1/f noise is not present. Demodulation of this signal shifts the results back to the desired frequency range of observation with the reduced noise floor of the higher frequencies. This method was found to improve sensitivities by nearly two orders of magnitude at 1 Hz and eliminated 1/*f* noise in the range from 1 Hz to 4 kHz. A mathematical model for low-finesse FP cavities is presented to support these results. This study suggests a relatively simple and efficient method for 1/f noise suppression and improving the device sensitivity of systems with an FP interferometer readout.

## 1. Introduction

Precision measurement of displacements and accelerations due to external forces are vital in a large array of scientific research and engineering. Highly sensitive acceleration measurements are necessary in a variety of fields, including inertial sensing [1], navigation [2], gravimetry [3], geodesy [4], and seismometry [5].

Such sensors have two main methods of readout, capacitive and optical. This manuscript focuses on a method for the removal of a 1/*f* noise. Interest in the field of opto-mechanics is growing, in part due to the high precision, sensitivity, and resolution of opto-mechanical sensors and their comparatively small foot-print and large bandwidth [6]. A detailed overview of the theory governing cavity opto-mechanics and opto-mechanical sensors can be found in [7] and, more recently, in [8]. A review of nano- and micro-scale opto-mechanical sensors is provided in [6], where three main types are discussed: passive opto-mechanical sensors, electro-opto-mechanical senors, and molecular-vibration-level sensors. Multiple examples of opto-mechanical sensors have been explored, with applications in displacement sensing [8], acceleration sensing [8,9], force sensing [8,10,11], magnetic field sensing [6,8,12], molecular sensing [6,13,14], quantum sensing [15], and even the search for dark matter [16,17]. Many of these functionalities rely on the high-precision displacement sensing capabilities of opto-mechanical sensors. The sensor used in this manuscript is predominantly for displacement and, ultimately, acceleration sensing.

Compact opto-mechanical resonators provide highly precise displacement measurements. The sensitivities of such systems depend on the mechanical resonator itself and the optical readout method. The latter measures the resonator’s motion, which is converted to acceleration using the resonator’s transfer function. An attractive displacement measurement method due to its excellent performance and simplicity is the use of fiber-based optical cavities [18,19,20,21,22,23,24,25]. In the configuration used here, the fiber ends are used as mirrors to create a low-finesse micro-cavity between the resonator’s test mass and frame. The motion of the test mass generates a signal that can be measured by a photo-detector (PD), which is first converted to displacement and then to acceleration. In its basic form, a *DC* measurement of the PD voltage is sufficient. However, this measurement is susceptible to low-frequency noise sources of different natures, e.g., 1/*f* electronics noise, PD dark current noise, or laser intensity fluctuations. The contribution of these noise sources can be minimized by using *AC* measurement techniques, as explored in this manuscript.

These techniques are especially important to mitigate the unavoidable 1/*f* noise present in electronic circuits at low frequencies, in our case, due to the transimpedance amplifier (TIA) of the PD. Where this 1/*f* noise is present, it limits the sensitivity of the device. Presented here is an *AC* method that suppresses this noise. Similarly, the low-frequency PD dark current noise and low-frequency laser intensity fluctuations are also minimized by this *AC* displacement readout.

Mitigation of noise in optical measurements varies widely depending on the source. Typical efforts focus on reducing laser source fluctuations [14]. The 1/*f* noise of optical origin is characterized by McDowell et al. in [26,27]. For optical setups, mitigation of 1/*f* noise can be performed via laser stabilization by locking the laser in an interferometric system to an external cavity via phase modulation, such as with the Pound–Drever–Hall method [28,29]. Alternatively, laser frequency modulation in a heterodyne interferometer can be used to eliminate 1/*f* noise [14]. For example, in [14], the laser frequency applied to the sensing arm of a heterodyne interferometer used for single particle detection was successfully up-converted, amplified, and down-converted to effectively remove 1/*f* noise, improving sensitivity by two orders of magnitude. The frequency up-conversion in this technique shifts the signal to a higher frequency range, where 1/*f* noise is not present, and it becomes limited by the optical background noise instead [14].

In this work, we implemented a similar, but alternative method for 1/*f* and low-frequency noise suppression, which, to our knowledge, has not previously been investigated. We used a mechanical modulation technique that shifts the signal content to higher frequencies before reaching the PD. This is then demodulated with a lock-in amplifier after the PD to shift the signal back to the desired measurement range. Instead of modulating the laser light itself though, as is common in other optically soured 1/f noise suppression techniques, we modulated the length of a micro-optical cavity using a piezoelectric transducer. This is similar though different from frequency modulating the laser’s light electric field, since [30,31]

(1) $$\frac{\delta \ell }{\ell }=\frac{\delta \nu }{\nu }$$

where *ℓ* and ν are the optical cavity length and laser frequency, respectively, and δ indicates fluctuations. Removing 1/*f* noise with this method allows for improved sensitivity without requiring extensive alterations to the setup. Of note, in contrast to other methods for 1/*f* noise reduction such PDH locking or up-conversions with modulation of the laser light itself, it requires no external electro-optic components such as electro-optic modulators (EOMs) for laser phase modulation or acousto-optic modulators (AOMs) for laser frequency modulation, as the laser light electric field is unaltered. It does not necessitate demodulation at high (GHz) frequencies, as is required with heterodyne techniques. Instead, its main component is a small piezoelectric transducer, keeping the system compact and relatively simple. It is a low-cost, low-power, and low-frequency scalable alternative with the potential to work in most homodyne readout systems that operate based on a change in optical path length.

This paper is organized as follows: in Section 2, the opto-mechanical resonator, i.e., the core of the accelerometer, is presented together with the fiber-based micro-optical cavity. Section 3 presents the displacement read-out technique using low-finesse optical cavities. Both the *DC* and *AC* methods are described. In Section 4, several noise sources are introduced and propagated through the system to determine their effect in the measurement. The experimental implementation and results are given in Section 5. We close with a summary and discussion in Section 6.

## 2. Mechanical Resonator

Opto-mechanical accelerometers consist of a mechanical resonator and an optical read-out. The resonator described here is a test mass held by leaf spring flexures, a configuration studied extensively for its simplistic, but highly sensitive design. Recent applications can be seen in [9,18,30,31,32,33] and demonstrate the geometry’s effectiveness. The resonator operates as a spring–mass-damped oscillator. This is a well-known and widely used model in physics and engineering, making the resonator design and analysis relatively straightforward. Derivation of the expected fundamental frequency and the displacement to the acceleration transfer function can be performed without complex modeling software. The dynamics of the resonator can be modeled as [34]:

(2) $$F/m=\ddot{x}+\Gamma_{\nu }\dot{x}+\omega_{0}^{2}\left(1+i\phi \right)x,$$

where *F* is an external force, *m* is the mass of the test mass, $\omega_{0}$ is the fundamental angular frequency of the resonator, $\Gamma_{\nu }$ is the velocity damping, ϕ are the mechanical losses of the material, and *x* is the dynamic variable representing the test mass motion when subjected to an external force *F*. Note that the response of the test mass and its transfer function for $\omega \ll \omega_{0}$ is simply [18,31]

(3) $$a_{\mathrm{ext}}=F/m=\omega_{0}^{2}x,$$

which indicates that the sensitivity of the resonator to external accelerations, $a_{\mathrm{ext}}$, is inversely proportional to $\omega_{0}^{2}$: the smaller the natural angular frequency, the larger the motion of the resonator when subjected to an external force.

**Figure 1:**
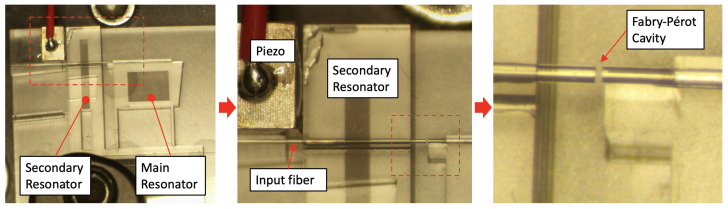
Left: The fused silica opto-mechanical sensor used in this study with labels identifying the resonator structures. Center: A zoomed-in view of the FPI and piezoelectric transducer location and setup on the sensor. Right: A further zoomed-in view of the 52 μm fiber Fabry–Pérot cavity located in the gap between the two resonators.

The mechanical resonator configuration used here is composed of two monolithic parallelogram resonators situated side by side on a common fused silica frame, each comprised of a test mass and two leaf spring flexures, as shown in Figure 1 [11,18,30,31,33,35]. The frame has outer dimensions of 10 mm × 17 mm × 20 mm. The main resonator, used as the actual accelerometer, has a fundamental frequency of 3.8 kHz. The displacement of the main resonator’s test mass is measured by a fiber-based low-finesse optical cavity or Fabry–Pérot interferometer (FPI), as described in Section 3.1. The displacement is converted to acceleration using the transfer function derived from Equation (2) or its simplified version, Equation (3). The secondary resonator is located beside the main one and is used here to implement the *AC* measurement technique, i.e., a cavity length modulation. The secondary resonator has a resonance frequency higher than the main one at 8.8 kHz. This ensures the motion of the main resonator plays the primary role in acceleration measurements since the secondary resonator is significantly stiffer and much less sensitive.

**Figure 2:**
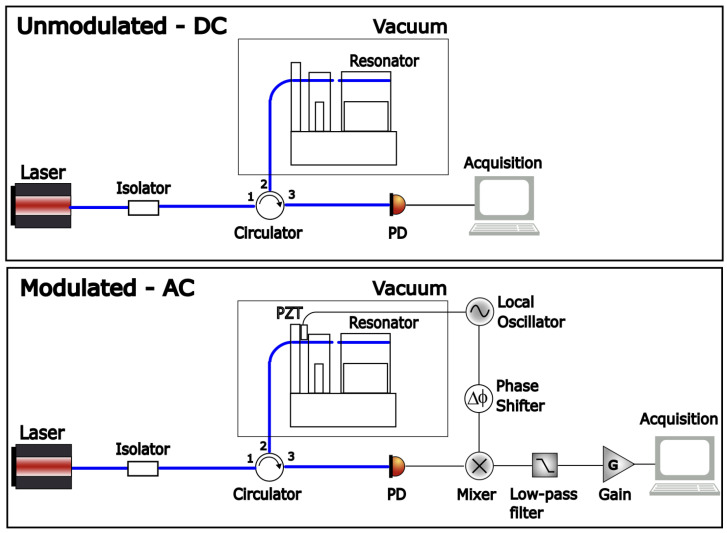
The schematic of the measurement setup for (**top**) the unmodulated *DC* system and (**bottom**) the modulated/demodulated *AC* system.

The optical cavity is formed between two bare fiber ends. One fiber end is glued to the secondary mechanical resonator, while the other is glued to the main resonator. Next to the secondary resonator, a piezoelectric transducer has been added to induce motion at high frequency in the secondary resonator. This motion translates into a length modulation of the optical cavity, an alternative to phase modulation on the light electric field, which is the common method for implementing laser frequency modulation in practice. The piezoelectric transducer has a resonance frequency around 500 kHz, well above the bandwidth of the main and secondary mechanical resonators. This large difference in frequency is necessary to avoid coupling the modulation into the main oscillator, which would excite the fundamental mode of the system. Ideally, excitation of this main mode should be solely caused by external accelerations. Additionally, high-frequency modulation is necessary to achieve a large bandwidth. Figure 1 shows the different parts of the opto-mechanical accelerometer, while Figure 2 shows a schematic of the displacement measurement techniques.

The two mechanical resonators are made from a single monolithic fused silica wafer, which has inherently low mechanical losses, ϕ, and, consequently, a high mechanical quality factor *Q* ($=\phi^{-1}$). High quality factors are necessary in order to reduce the fundamental thermal noise limit of the mechanical resonators [34]. In our case, we measured in vacuum (2.2 μTorr) a modest quality factor of around 770. This comparatively low *Q* is the result of surface loses. Nevertheless, the thermal noise for such a quality factor expressed in terms of displacement is about 0.05 fm/$\sqrt{\mathrm{Hz}}$ at 1 Hz and does not limit our investigations presented here.

## 3. Low-Finesse Micro-Optical Cavity Displacement Sensor

Fabry–Pérot interferometers are very well suited to measure the displacement of the mechanical resonator’s test mass with high precision [18,19,20,21,22,23,24,25]. In our case, we used two optical fibers cleaved to a near-zero-degree angle. The cleaved edges of the fibers act as flat mirrors with low reflectivity (∼4%), forming a low-finesse optical cavity. One of the fibers is adhered to the test mass of the main resonator with UV curing adhesive. The other spans across and is adhered to the test mass of the secondary resonator in the opposing direction, as shown in the pictures in Figure 1 and the schematic in Figure 2. Light is brought to the cavity using an optical circulator. The laser light is coupled with port 1 and transmitted to port 2, which is connected to the input fiber of the cavity. The light reflected from the cavity is coupled back with the circulator and transmitted to port 3, where a photodetector measures the signal generated from the cavity. A 1550 nm wide tunable Newport laser was used as the light source. Wide-band tunability is necessary to operate with μm long optical cavities.

**Figure 3:**
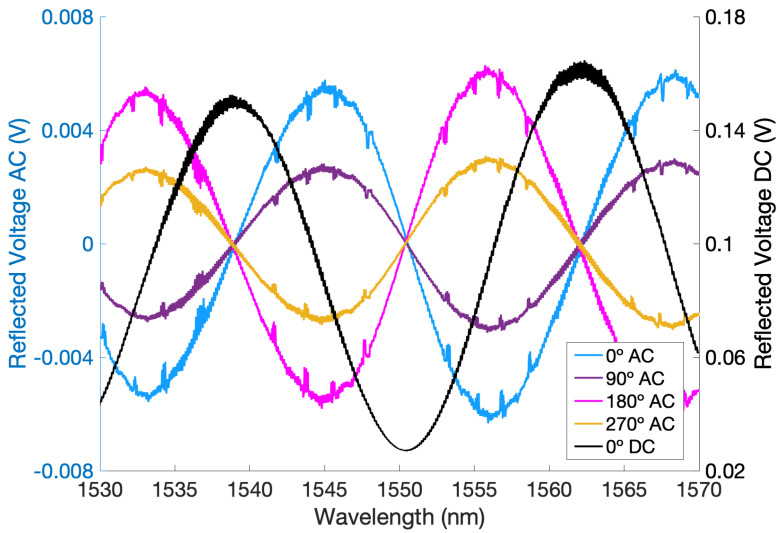
Wavelength scans of the Fabry–Pérot cavity. In-air low-pass-filtered wavelength scans of the demodulated *AC* signal with various phase shifts (blue voltage scale on the left) plotted alongside the DC signal, which is not yet demodulated (black trace with black voltage scale on the right) for the comparison of slopes and working point locations. The cavity can be characterized using the black *DC* trace, which appears the same as the unmodulated *DC* wavelength scans and where γ=85.36%, $\ell_{0}=51.73$ μm, FSR=2.9 THz, and F=2.36. Working point locations are at the half-maximum, where the curve is approximately linear. It is clear that the working point location shifts about 5 nm between the DC and AC cases. In *AC*, the maximum slopes occurs at 0° and 180° phase shifts.

An important parameter for any optical cavity is the free spectral range, FSR =c/2ℓ, where *c* and *ℓ* are the speed of light and the cavity’s length, respectively. In our case, the cavity length is about 50 μm (see Figure 3), which is equivalent to an FSR of 3 THz. This length is a compromise. A much shorter cavity implies an extremely large FSR, which reduces sensitivity in low-finesse cavities and requires an exceptionally large range of tunability in the laser. Likewise, a much longer cavity degrades the coupling of light due to the plano–plano configuration, increasing optical losses.

Another important parameter defining an optical cavity is the finesse F. For low-reflectivity mirrors, $F=\pi \sqrt{R}/\left(1-R\right)=FSR/FWHM$, where R is the reflectivity of the mirrors and FWHM is the full-width at half-maximum of the resonant dips. Though high-finesse cavities create a more sensitive readout, the use of a low-finesse cavity reduces the sensitivity to misalignments. It also eases the displacement measurement read-out since the operating working point is much wider than in a high-finesse cavity, where the transmission dips described in Section 3.1 are much sharper and span fewer wavelengths. The FSR of the cavity was measured to be about 2.9 THz by a wavelength scan from 1530 nm to 1570 nm (shown in Figure 3). This corresponds to a cavity length of 51.7 μm. The visibility (or contrast) was about 85%, and the finesse was about 2.4 (indicating our reflectivity R was slightly higher than the anticipated 4% for un-coated glass, where F=0.65).

### 3.1. DC Measurement Technique

The simplest displacement measurement technique using the FPI is sketched in the top schematic of Figure 2. The PD in the third port of the circulator measures the response of the cavity. The output signal of the PD depends on the laser frequency/wavelength and the actual cavity length. For a Fabry–Pérot cavity, the light is transmitted into the cavity when the laser’s wavelength is in resonance with the cavity length according to the resonance condition 2ℓ=nλ, where *n* is an integer [28]. This results in a decrease in reflected light when the system is in resonance, i.e., resonant dips in the voltage output. Movement of the test mass results in a change in the length of the cavity and, therefore, a change in the resonance condition. For a low-finesse cavity, the response resembles a sine wave, as shown in Figure 3. The exact response is an Airy function. The PD photocurrent intensity from the third port of the circulator is [18,30,32,33,36]

(4) $$I_{r}\left(\ell ,\lambda \right)=2RI_{0}\left(\frac{1-\cos\frac{4\pi }{\lambda }\ell }{1+R^{2}-2R\cos\frac{4\pi }{\lambda }\ell }\right)≃2RI_{0}\left(1-\cos\frac{4\pi }{\lambda }\ell \right),$$

where R≈4% is the reflectively of the cavity mirrors (the cleaved fibers), $I_{0}$ is the initial *DC* photocurrent, which is proportional to the optical power at the input of the cavity (neglecting insertion losses in the circulator), λ is the laser’s wavelength, and *ℓ* is the optical cavity length. The approximation is valid for small *R* values. The response given by Equation (4) is clearly visible in Figure 3 for a wavelength scan from 1530 to 1570 nm (assuming constant *ℓ*).

The optimal laser frequency (or wavelength) is the one that maximizes the current change for a given cavity length. The steepest current/voltage slope in this instance ($m_{\ell }$) is readily calculated from Equation (4):

(5) $$m_{\ell }=\max\left\{\frac{\partial I_{r}}{\partial \ell }\right\}=\frac{8\pi RI_{0}}{\lambda_{m}}.$$

This occurs for $\lambda_{m}=8\ell /n$ with n an odd integer. In our case, n is near 260 since we are operating at $\lambda_{m}∼1550$ nm and ℓ∼50 μm. Thus, the distance between two maxima slopes is roughly $16\ell /n^{2}≃10$ nm. In practice, the working point is found experimentally from a wavelength scan measurement, as shown in Figure 3. In this case, the steepest slope is also calculated from Equation (4), but with respect to a change in wavelength ($m_{\lambda }$). For $\lambda =\lambda_{m}$, this is

(6) $$m_{\lambda }=\max\left\{\frac{\partial I_{r}}{\partial \lambda }\right\}=-\frac{8\pi RI_{0}\ell }{\lambda_{m}^{2}}.$$

In Figure 3 (*DC*), these slopes occur at around 1535 nm, 1545 nm, and 1555 nm, i.e., in intervals of 10 nm, as expected. Setting the laser at any of these wavelengths maximizes the sensitivity of the displacement read-out since this is where the most drastic change in current (voltage) occurs for a given cavity length change. Thus, once the laser wavelength is *fixed*, cavity length changes, i.e., test mass motion, can be inferred from the photodetector measurement:

(7) $$\delta \ell =\frac{1}{m_{\ell }}\delta I_{r}=-\frac{\ell }{\lambda_{m}}\frac{1}{m_{\lambda }}\delta I_{r}$$

where $m_{\lambda }$, *ℓ*, and $\lambda_{m}$ are known from the wavelength scan (Figure 3) and $\delta I_{r}$ is the photo-detector signal [18,31,33]. For the optimal wavelength $\lambda_{m}$ and the assumption of small length changes, the *DC* current becomes:

(8) $$I_{r}≃2RI_{0}+2RI_{0}\frac{4\pi }{\lambda_{m}}\delta \ell .$$

### 3.2. AC Measurement Technique

*AC* measurement techniques can be implemented in our setup in order to mitigate 1/*f*, dark current, and laser intensity noise. The method relies on the length modulation of the optical cavity by means of a piezoelectric transducer at very high frequency (well above the bandwidth of the main mechanical resonator), as described in Section 2. In this section, we give a mathematical description, which is later used to investigate the noise sources and the way they couple with the measurement.

In general, the signal at the PD for a given *ℓ* and laser wavelength is

(9) $$I_{r}=2RI_{0}\left(1-\cos\left[\frac{4\pi }{\lambda }\left(\ell_{0}+\Delta \ell \right)\right]\right)$$

where Δℓ indicates changes from the nominal value $\ell_{0}$. Here, Δℓ is the sinusoidal signal generated by the piezoelectric transducer at around 500 kHz, which modulates the cavity length. Thus, Equation (9) can be written as

(10) $$I_{r}=2RI_{0}\left(1-\cos\frac{4\pi }{\lambda }\ell_{0}\left(1+\beta \sin\omega_{m}t\right)\right)$$

where β is the modulation depth ($=\Delta \ell /\ell_{0}$) and $\omega_{m}$ the angular frequency modulation, respectively. This equation can be expanded to

(11) $$\begin{array}{ccc} I_{r} & = & 2RI_{0}\left[1-\cos\left(\frac{4\pi }{\lambda }\ell_{0}\right)\sum_{k=-\infty }^{\infty }J_{k}\left(\frac{4\pi }{\lambda }\ell_{0}\beta \right)\cos\left(k\omega_{m}t\right)\right.\\ & & \left.+\sin\left(\frac{4\pi }{\lambda }\ell_{0}\right)\sum_{k=-\infty }^{\infty }J_{k}\left(\frac{4\pi }{\lambda }\ell_{0}\beta \right)\sin\left(k\omega_{m}t\right)\right], \end{array}$$

where $J_{k}$ is a Bessel function of the first kind. The modulated signal $I_{r}$ is later demodulated at $\omega_{m}$ by mixing it with a phase-shifted version of the signal driving the piezoelectric transducer. Therefore, the only terms of the Bessel expansion remaining after demodulation are those for k=±1 (we also neglect the DC term). Thus,

(12) $$I_{r}^{\omega_{m}}=4RI_{0}J_{1}\left(\frac{4\pi }{\lambda_{0}}\ell_{0}\beta \right)\sin\left(\frac{4\pi }{\lambda }\ell_{0}\right)\sin\left(\omega_{m}t\right),$$

where the cosine terms vanish because Bessel functions of the first kind with an odd order and sine functions both have odd parity, while cosine functions have even parity. The signal in Equation (12) after demodulation and low-pass filtering is

(13) $$I_{r}^{\mathrm{demod}}=2RI_{0}J_{1}\left(\frac{4\pi }{\lambda }\ell_{0}\beta \right)\sin\left(\frac{4\pi }{\lambda }\ell_{0}\right),$$

which is the same found for the sinusoidal term in the *DC* case (cf. Equation (4)), except it is shifted by π/2, and the amplitude is $J_{1}$ smaller. The phase difference has no effect at all on the performance; it just means the optimal wavelengths in the *DC* and *AC* cases are not the same. This behavior is shown in Figure 3: the black trace is the *DC* signal prior to demodulation in a modulated measurement (which appears the same as an unmodulated wavelength scan), while the others are *AC* measurements with different demodulation phase shifts. The smaller scaling factor indicates a degradation of sensitivity, i.e., a shallower slope $m_{\ell }$ (and $m_{\lambda }$). While this is a disadvantage of the method, the benefits of avoiding low-frequency noise outweigh the sensitivity reduction, and if necessary, it can be compensated by increasing the laser power. Note the maximum value of $J_{1}\left(x\right)$ is 0.58 (at *x* = 1.84). Thus, the optimal modulation depth is found as

(14) $$\frac{4\pi }{\lambda }\ell \beta_{\mathrm{opt}}=1.84,$$

which for λ = 1550 nm and *ℓ* = 50 μm yields $\beta_{\mathrm{opt}}=0.0045$, i.e., Δℓ≃0.2 μm.

## 4. Noise Analysis

In this section, we analyze the coupling of several noise sources in the displacement read-out both in the *DC* and *AC* cases.

### 4.1. Dark Current and Transimpedance Amplifier Noise

Typically, the current is amplified and converted to voltage with a transimpedance amplifier (TIA). For the *DC* case, the voltage at the TIA’s output is simply

(15) $$V_{\mathrm{DC}}≃G\left[2RI_{0}\left(1+\frac{4\pi }{\lambda_{m}}\delta \ell \right)+i_{\mathrm{dark}}+n_{\mathrm{TIA}}\right],$$

where *G* is the transimpedance gain, $i_{\mathrm{dark}}$ is the PD’s dark noise, and $n_{\mathrm{TIA}}$ is the TIA’s noise referring to the input. Thus, the noise converted to length and expressed in power spectral density (PSD) is:

(16) $$S_{\mathrm{npd}}^{\mathrm{DC}}\left(\omega \right)=\frac{1}{m_{\ell }^{2}}\left[S_{\mathrm{dark}}\left(\omega \right)+S_{\mathrm{TIA}}\left(\omega \right)\right]$$

where it is clear any 1/*f* noise in the TIA or PD will appear as noise in the displacement measurement. As expected, the noise is inversely proportional to the slope $m_{\ell }^{2}$ (in terms of PSD).

The *AC* case is slightly different since the amplification takes place before the mixing and low-pass filtering. Thus, the TIA output is mixed with $\omega_{m}$ and low-pass filtered. Expressed as a voltage, this is:

(17) $$V_{\mathrm{AC}}≃\mathrm{LPF}\left\{G\left[2RI_{0}+4RI_{0}J_{1}\frac{4\pi }{\lambda_{m}}\delta \ell \sin\omega_{m}t+i_{\mathrm{dark}}+n_{\mathrm{TIA}}\right]\sin\omega_{m}t\right\},$$

where LPF stands for low-pass filter. After mixing and low-pass filtering, we are left with the following noisy terms:

(18) $$S_{\mathrm{npd}}^{\mathrm{AC}}\left(\omega \right)=\frac{1}{{\left(J_{1}m_{\ell }\right)}^{2}}\left[S_{\mathrm{dark}}\left(\omega -\omega_{m}\right)+S_{\mathrm{TIA}}\left(\omega -\omega_{m}\right)\right],$$

where it is clear that the dark current and transimpedance amplifier noise at $\omega_{m}$ is down-converted to ω, while the 1/*f* noise is up-converted to $\omega_{m}$. For the optimal modulation depth β=0.0045 where $J_{1}=0.58$, the minimum possible PD noise for the presented cavity is near 0.2 pm/$\sqrt{\mathrm{Hz}}$ when expressed as an amplitude spectral density (ASD), the square root of the PSD. This is found using Equation (18), where the total of the PSD terms in brackets is determined using the PD data sheet [37] and the measured working point voltage at the PD.

### 4.2. Laser’s Intensity and Frequency Fluctuations

The intensity noise for the *DC* case couples with the length noise in the same manner as the dark current and TIA noise. In terms of the PSD, it is:

(19) $$S_{\mathrm{nrin}}^{\mathrm{DC}}\left(\omega \right)=\frac{{\left(2R\right)}^{2}}{m_{\ell }^{2}}S_{I}\left(\omega \right)={\left(\frac{\lambda_{m}}{4\pi }\right)}^{2}\frac{S_{I}\left(\omega \right)}{I_{0}^{2}},$$

where $S_{I}\left(\omega \right)/I_{0}^{2}$ is the relative intensity noise (RIN) of the laser, which increases rapidly towards lower frequencies. Our laser exhibits RIN around $10^{-4}$ 1/$\sqrt{\mathrm{Hz}}$ at 1 Hz, which is equivalent to 10 pm/$\sqrt{\mathrm{Hz}}$. The laser intensity noise in the *AC* scheme is also similar to the dark current and TIA noise. Its contribution, in terms of the PSD, is:

(20) $$S_{\mathrm{nrin}}^{\mathrm{AC}}\left(\omega \right)={\left[\frac{2(2R)}{J_{1}\left(\frac{4\pi }{\lambda }\ell_{0}\beta \right)m_{\ell }^{2}}\right]}^{2}S_{I}\left(\omega -\omega_{m}\right)={\left[\frac{\lambda_{m}}{4\pi }\frac{2}{J_{1}\left(\frac{4\pi }{\lambda }\ell_{0}\beta \right)}\right]}^{2}\frac{S_{I}\left(\omega -\omega_{m}\right)}{I_{0}^{2}},$$

where, now, the contribution of RIN in the low-frequency band is due to the RIN level at $\omega_{m}$, which is typically much smaller than the RIN at low frequencies. At $\omega_{m}≃500$ kHz, it is about $5\times 10^{-7}$ 1/$\sqrt{\mathrm{Hz}}$, which is equivalent to 0.5 pm/$\sqrt{\mathrm{Hz}}$. The RIN contribution decreases by nearly two orders of magnitude with the modulated readout scheme.

Finally, frequency fluctuations of the laser couple identically for the *DC* and *AC* cases. In the PSD, they are:

(21) $$S_{n\nu }^{\mathrm{DC},\mathrm{AC}}\left(\omega \right)={\left(\ell_{0}\frac{\lambda_{m}}{c}\right)}^{2}S_{\nu }\left(\omega \right),$$

which, in our case, has a very small contribution, 20 fm/$\sqrt{\mathrm{Hz}}$, when considering the laser frequency noise of the 1550 nm tunable laser is about ∼100 kHz/$\sqrt{\mathrm{Hz}}$ at 1 Hz.

The total noise in the system in terms of the PSD is simply the sum of the noise sources described above. The ASD is simply the square root of this PSD sum. For the *AC* case, the total displacement noise is, therefore:

$$ASD_{AC}=\sqrt{S_{n}^{AC}}=\sqrt{S_{\mathrm{npd}}^{\mathrm{AC}}\left(\omega \right)+S_{\mathrm{nrin}}^{\mathrm{AC}}\left(\omega \right)+S_{n\nu }^{\mathrm{AC}}\left(\omega \right)}.$$

This calculated *AC* readout agrees well with the experimental results shown in the following section, Section 5.

## 5. Experimental Implementation and Results

The experimental results using both the *DC* and *AC* measurement techniques are presented here. The tests were carried out in vacuum conditions, near 3 μTorr. The schematic setup of these configurations is shown in Figure 2. One might recognize the modulated configuration as similar to that of the Pound–Drever–Hall (PDH) laser frequency locking scheme [28] except the cavity length rather than the laser light phase is modulated and the mixed signal output is read directly instead of being fed back to the laser source for a stabilizing feedback loop.

The resonant modes of the piezoelectric transducer attached to the secondary input resonator were found by scanning frequencies well above the resonance frequency of the main fused silica resonator and around the documented piezoelectric transducer resonance to maximize the modulation depth β. The piezoelectric transducer resonance frequency was found at 523 kHz, and thus, we used this frequency for the *AC* measurement technique. Note that this frequency is well above the fundamental mode of the main resonator in the sensor and, thus, minimizes cross-talk. The amplitude of the sinusoidal modulation was 1 Vpp, which yielded a modulation depth of β=0.053 and $J_{1}=0.124$. Since the difference in the oscillating term of the current between the *AC* and *DC* measurements is simply a factor of $J_{1}$, these were calculated by comparing the ratio between the measured *AC* and *DC* slopes $m_{\lambda }$ and the ratio between the *AC* and *DC* current equations. In the *AC* measurement, the signal from the photo-detector was demodulated by the same oscillator driving the piezoelectric transducer. The phase was adjusted in order to maximize the signal during a wavelength scan. The theoretical optimal phase is zero degrees, which agrees well with the experimental results shown in Figure 3. This figure also shows other phase shifts equivalent to the optimal. In the measurements presented here, a phase shift of 180° was used, which has the same slope, but with opposite sign. The mixed signal was then passed through a low-pass filter with a 10 kHz corner frequency to eliminate the terms at multiples of $\omega_{m}$. The mixing and low-pass filtering for demodulation were performed digitally in a commercial digital signal processing unit (Moku:Lab), where a gain of 20 dB was also applied to amplify the signal after the low-pass filter.

Because the demodulation shifts the location of the maximum slope according to the unmodulated signal’s derivative (Figure 3), the response signal shifts by 90°, and thus, the operating wavelength also must be shifted for the modulated measurements. This is derived mathematically in Section 3. To ensure the operation at the location of maximal sensitivity, a wavelength scan between 1530 nm and 1570 nm was taken before every measurement for both the modulated and unmodulated configurations. The operating wavelength and maximum slope $m_{\lambda }$ of the transmission dip was then recalculated and adjusted according to these wavelength scan results.

Wavelength scans were performed before both the *DC* and *AC* setup to find and set the optimal wavelength for each. For the results presented here, the *DC* measurements were performed at $\lambda_{m}=1555.7$ nm, while the *AC* ones were taken at $\lambda_{m}=1561.1$ nm. The 5 nm difference agrees with the expected theoretical one.

**Figure 4:**
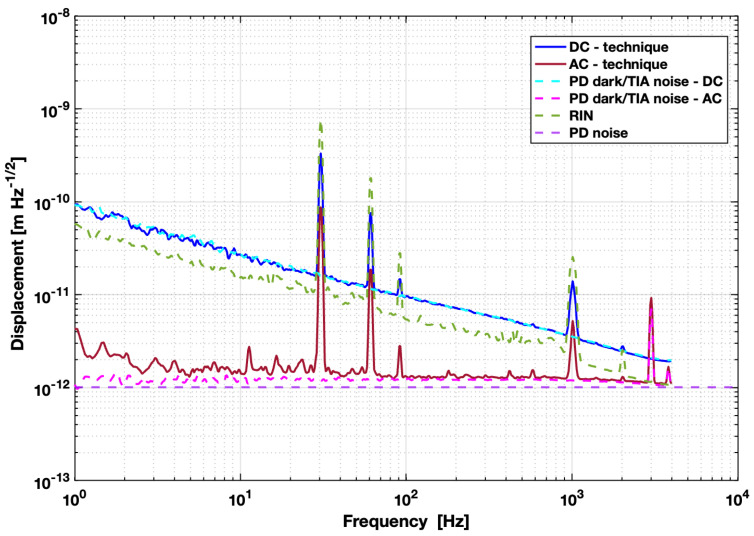
The amplitude spectral density of a 5-minute unmodulated (blue) measurement and a 5-minute modulated and demodulated (maroon) measurement between 1 Hz and 10 kHz, where 1/f noise is observed. Measurements were taken in vacuum near 3 μTorr. Also shown are 5-minute measurements of RIN (dashed green) and PD noise (dashed cyan and magenta). Additionally, the dashed purple line shows the theoretical PD noise based on its data sheet [37]. We note the alignment of the displacement noise with the measured PD noise.

The motion displacement results are shown in Figure 4 in terms of the ASD, i.e., in units of m/$\sqrt{\mathrm{Hz}}$. The frequency range spans from 1 Hz to 5 kHz, the range where the *AC* technique showed a significant effect. The displacement sensitivity was 90 pm/$\sqrt{\mathrm{Hz}}$ at 1 Hz for the *DC* technique and 4 pm/$\sqrt{\mathrm{Hz}}$ (white noise) at 1 Hz for the *AC* technique. It is clear that the *AC* modulation technique effectively suppressed the 1/*f* noise present in the *DC* case and, thus, provided significant improvement compared to the unmodulated results.

Figure 4 also shows the apportioning of the noise sources described in Section 4. These include measurements for photo-detector noise with (*AC*) and without (*DC*) modulation (magenta and cyan) and RIN (green). The RIN was measured by setting the operational wavelength at the voltage peak, where fluctuations are expected to have little effect, due to the zero slope (green). The dark current and TIA noise are also plotted (dashed cyan and dashed magenta). These were obtained by capping the PD so no light hit the sensor and repeating the measurement schemes with and without modulation. Additionally, the theoretical photo-detector noise according to its data sheet is also plotted (purple) [37].

At the working point, a voltage of 100 mV, or a power of roughly 60 μW, was observed before demodulation. Applying this value to the theoretical *AC* model presented in Section 4.1 by using Equations (5) and (18) with our cavity setup and $J_{1}=0.124$, we found an expected PD noise floor of roughly 0.82 pm/$\sqrt{\mathrm{Hz}}$. Adding this to the theoretical values for RIN and laser frequency noise in Section 4.2, we expected a total *AC* noise ASD of about 1.35 pm/$\sqrt{\mathrm{Hz}}$. This calculated *AC* readout agrees well with the experimental measurement results shown in Figure 4, where the ASD of the modulated measurement (maroon trace) is between 1 and 2 pm/$\sqrt{\mathrm{Hz}}$ for the majority of frequencies and the PD noise is the dominant contribution (dashed magenta trace).

From this, it is clear that the PD noise floor aligns well with the measured noise floor of our system, indicating that PD noise was the main limiting factor in the setup, rather than RIN or laser frequency noise, which are both anticipated to be lower than the measured results. That the PD noise creates the limiting 1/f trend is consistent with [26], which found that 1/f noise is photo-detector dependent. The laser frequency noise is not plotted in Figure 4, as it was significantly lower than all other noise sources at 8 fm/$\sqrt{\mathrm{Hz}}$ at 1 Hz. Similar displacement sensitivity results found with in-air measurements also suggest gas damping and *Q*, which should improve in vacuum, are not limiting in this instance either. The calculation of the theoretical thermal acceleration noise floor for a *Q* of 770 gives a maximum acceleration on the order of 70 nm/$s^{2}\sqrt{\mathrm{Hz}}$. Conversion of the measured displacement values puts an acceleration limit on the order of 1 mm/$s^{2}\sqrt{\mathrm{Hz}}$, far above this theoretical value, re-enforcing the notion that *Q* is not limiting in this instance.

As shot noise is typically limiting at higher frequencies, using a more sensitive photo-detector would be beneficial for improving the readout sensitivity of the device when combined with the modulation scheme presented since the PD noise is the current limit. In addition, better optimization of the modulation depth β could be applied to maximize the AC slope and improve sensitivity, as an optimized Bessel function scale factor $J_{1}=0.58$ resulted in a theoretical noise floor roughly a factor of five better than that with $J_{1}=0.124$. Note, from our modulation depth value, a displacement of about 2.7 μm was attained, larger than the documented maximum due to the piezoelectric transducer being driven at its resonance. According to Equation (1), this means we achieved deep modulations of the optical cavity frequency in the order of 1 THz at a notably high modulation frequency of 523 kHz, by using a piezoelectric transducer acting directly on the cavity length. We would not be able to achieve this with direct frequency modulation of the laser or with EOMs.

As this is an opto-mechanical system, fluctuations of the environmental temperature will induce small length changes within the cavity and can affect the calibrated slope over time by introducing a gain error. To avoid this, a slope calibration should be performed periodically or, most ideally, before each measurement. For the long term, ongoing measurements, power monitoring, and stabilization can help minimize the effect. Likewise, the materials and adhesives used in the system will be limiting at temperature extremes. Of note, the UV-curing adhesive used to attach the fibers and piezoelectric transducer in this study could fail at cryogenic temperatures if not rated for such environments. Other environmental factors, such as electromagnetic interference or mechanical vibrations, will only hinder the method if they occur at the same frequency as the modulation (here, 523 kHz). As such, piezoelectric transducer modulation at frequencies of known electric or mechanical vibrations in the area of operation should be avoided. A more thorough investigation in future studies of the effects these and other environmental factors have on the stability of the method would provide more insight into how they might influence the results of the technique.

## 6. Conclusions

In this work, we have investigated a method for the mitigation of 1/f noise of optical origin in a displacement measurement with the use of an *AC* modulation technique that, to our knowledge, has not been previously explored. This technique implements a mechanical modulation of a fiber Fabry–Pérot cavity on an opto-mechanical sensor to up-convert the readout signal and uses a post PD demodulation to shift the signal back to the desired measurement range, a method analogous to, but different from laser frequency and phase modulation. A theoretical interpretation of this method also indicates the mitigation of low-frequency laser intensity fluctuations. We present this mathematical model with the parameters of our low-finesse cavity, which supports and confirms the measured results. Multiple noise sources were measured in addition to the *AC* and *DC* schemes, including the dark current and TIA noise in the PD, RIN, and laser frequency noise. The photo-detectors were observed as the primary limiting noise source in the system and are the typical cause of 1/f noise in optical readouts. With the *AC* method, this 1/f noise in the displacement measurement was effectively removed resulting in a frequency-independent noise between 1 Hz and 5 kHz. Sensitivities improved by nearly two orders of magnitude near 1 Hz from 90 pm/$\sqrt{\mathrm{Hz}}$ to 4 pm/$\sqrt{\mathrm{Hz}}$.

The technique has the advantage of being compact and low cost with no need for external electro-optical components such as EOMs and AOMs. It reaches a meaningful modulation depth in a very short cavity in a simple and inexpensive way. When optimized with the piezoelectric transducer used, it is capable of achieving cavity frequency modulations up 1 THz with a low mechanical modulation near 500 kHz, an amplitude and frequency not attainable with other methods. This modulation scheme provides an effective and relatively simple technique for improving displacement sensitivity. It can potentially be expanded to most homodyne readout systems that function due to optical path length changes, including displacement, refraction, and spectroscopy measurements. It is scalable to larger and smaller sizes, making it a potential candidate for integration in photonic systems such as micro-opto-electro-mechanical-systems (MOEMSs). Future studies with these other readout methods would expand on the results presented here.

## Data Availability

The original contributions presented in the study are included in the article; further inquiries can be directed to the corresponding author(s).

## Abbreviations

The following abbreviations are used in this manuscript:

ASD	amplitude spectral density
FP	Fabry–Pérot
FPI	Fabry–Pérot interferometer
FSR	free spectral range
FWHM	full-width at half-maximum
PD	photo-detector
PSD	power spectral density
TIA	transimpedance amplifier
